# A nitrification bioreactor applied solely with ammonium and inorganic C maintains a highly diverse bacterial and archaeal community even after nine years

**DOI:** 10.1007/s10532-026-10288-9

**Published:** 2026-07-06

**Authors:** Luc Dendooven, Saúl López-Vázquez, Valentín Pérez-Hernández, Mario Hernández-Guzmán, Nina M. Montoya-Ciriaco, Marco Luna-Guido, Frederic Thalasso, Yendi E. Navarro-Noya

**Affiliations:** 1 Biotecnología y Bioingeniería, Centro de Investigación y de Estudios Avanzados del Instituto Politécnico Nacional, Mexico City, Mexico; 2 Laboratorio de Ecología del Suelo, Cinvestav, 07360 Mexico City, Mexico; 3 Laboratorio de Bioprocesos, Cinvestav, 07360 Mexico City, Mexico; 4 Laboratorio de Interacciones Bióticas, Centro de Investigación en Ciencias Biológicas, Universidad Autónoma de Tlaxcala, Tlaxcala, Mexico

**Keywords:** Archaeal and bacterial community, High-throughput shotgun analysis, Nitrifying reactor, Genes involved in the N cycle, Microbiome of engineered environments

## Abstract

The archaeal and bacterial community was determined in a continuous aerobic nitrifying reactor maintained under similar conditions for > 9 years, applied solely with ammonium as energy source and carbonate as C source. The high-throughput shotgun analysis revealed 4483 bacterial and 245 archaeal species that thrived on the metabolites provided by the autotrophic nitrifying population, mostly the ammonia oxidizing bacteria *Nitrosomonas europaea* and archaea *Candidatus* Nitrosocosmicus exaquare, and the nitrite oxidizing bacteria *Nitrobacter winogradskyi* and *N. hamburgensis*. *Candidatus* Nitrospira inopinata capable of oxidizing ammonia to nitrate was also detected. Although the reactor was supplied with sufficient O_2_, the anaerobic bacteria *Candidatus* Kuenenia stuttgardiensis capable of oxidizing ammonium to dinitrogen gas using nitrite as the electron acceptor under anoxic conditions (anammox) and four archaeal genera of the strict Methanobacteriaceae were detected in the reactor. A wide range of genes encoding for the different processes involved in N cycling were detected including the *hzo* gene encoding for the hydrazine oxidoreductase participating in the anaerobic anammox process. It was found that a bioreactor applied only with ammonium as energy source and maintained for > 9 years under steady state conditions contained a highly diverse bacterial and archaeal population and a wide range of metabolic processes related to the N cycle, which has not been reported before. This provides us with comprehensive insights into the dynamics of microbial communities in these types of systems.

**Graphical abstract:**

**Figure:**
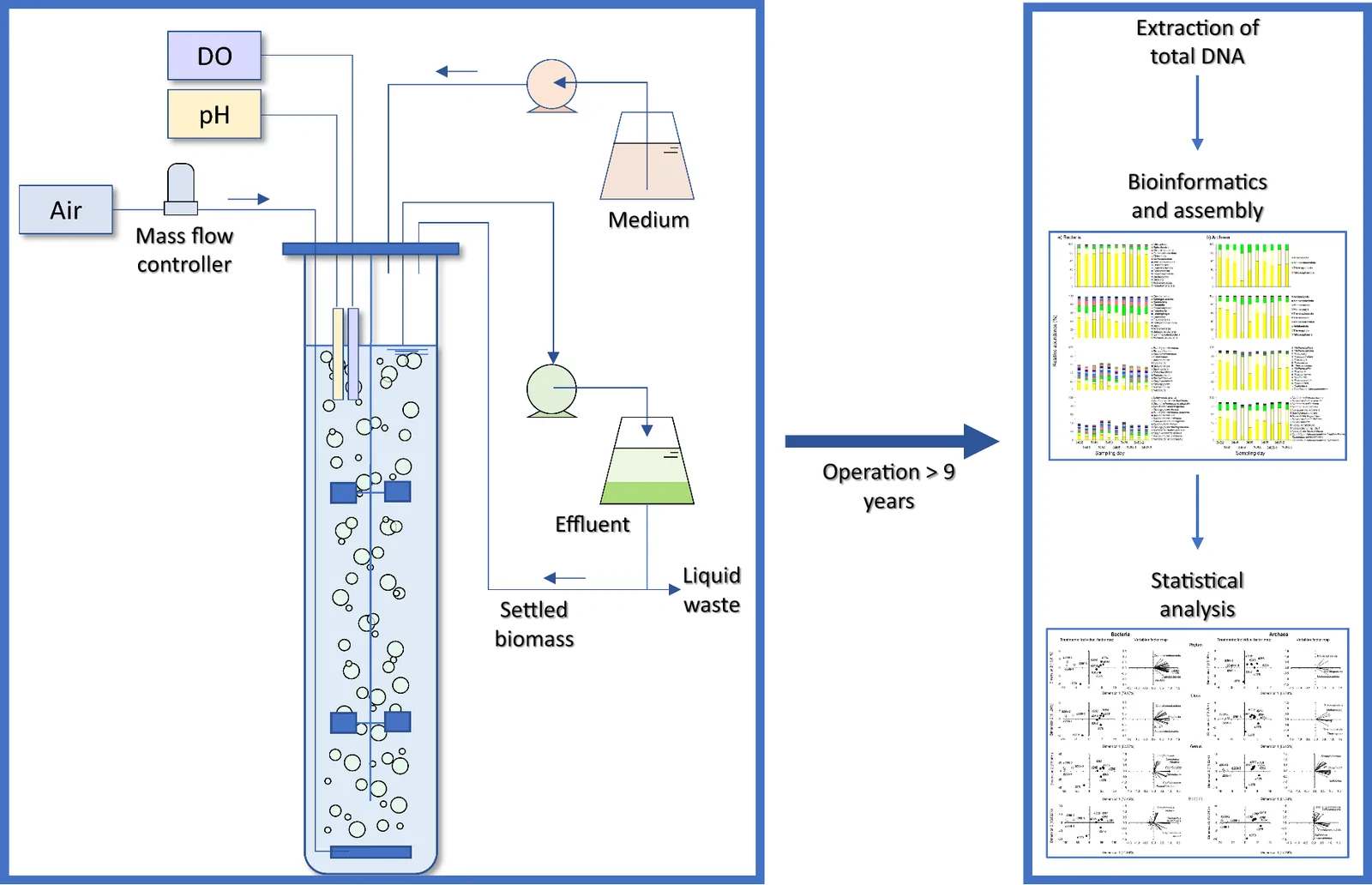

**Supplementary Information:**

The online version contains supplementary material available at https://doi.org/10.1007/s10532-026-10288-9.

## Introduction

Several processes are involved in the nitrogen (N) cycle in any given ecosystem. Nitrogen fixation by bacteria converts inert dinitrogen (N_2_) to assimilable ammonium (NH_4_^+^) while NH_4_^+^ is oxidized to nitrite (NO_2_^−^) and then nitrate (NO_3_^−^) by nitrifiers. Under anaerobic conditions, NO_3_^−^ can serve as electron acceptor and be reduced to nitrous oxide (N_2_O) and/or N_2_. Heterotrophic aerobic denitrifiers, such as *Klebsiella variicola* and *K. pneumoniae* might play a role in the removal of NO_3_^−^ from wastewater (Feng et al. 2018).

Nitrification is normally a two-step process and depends on the synergic activity of two different microbial groups, i.e., ammonia oxidizers and nitrite oxidizers, although recently bacteria have been described and isolated that can oxidize NH_4_^+^ to NO_3_^−^ (Kits et al. 2017). First, ammonia (NH_3_) is oxidized by ammonia-oxidizing bacteria (AOB) or ammonia-oxidizing archaea (AOA) to hydroxylamine (NH_2_OH) and further to NO_2_^−^. Nitrite is then oxidized by nitrite-oxidizing bacteria (NOB) to NO_3_^−^. Nitrifiers are mostly autotrophic bacteria that use the oxidation of NH_4_^+^ or NO_2_^−^ to generate energy and use inorganic C as substrate, but heterotrophic nitrification has been detected in a wide range of bacteria (e.g., *Pseudomonas tolaasii* Y-11) (He et al. 2016).

Nitrification is a critical process in wastewater treatment as it facilitates biological nitrogen removal, thereby preventing eutrophication caused by the overgrowth of algae and cyanobacteria (Ge et al. 2015). Nitrifying organisms oxidize NH_4_^+^ to NO_3_^−^, while denitrifiers subsequently reduce NO_3_^−^ to gaseous nitrogen forms (N_2_O and N_2_), effectively removing nitrogen with minimal sludge biomass production (Bhattacharya and Mazumder, 2021). However, slow-growing nitrifiers are sensitive to a wide range of environmental conditions, including dissolved oxygen (DO), inhibitory organic compounds, temperature and pH, which often leads to the inhibition of the oxidation of NH_4_^+^ to NO_3_^−^ (Chen et al. 2017). Consequently, nitrification is often the rate-limiting step in the removal of N from wastewater.

There are several studies that characterized nitrifying consortia, i.e., bacteria and archaea, in activated sludge or biofilms (aerated filters) (e.g., Al-Ajeel et al. 2022). For instance, Shukla and Ahammad (2022) reported that Pseudomonadota, Planctomycota, Chloroflexota and Actinomycetota were the dominant phyla in conventional activated sludge process with a modified trickling filter for urban sewage treatment with a large variation of nitrogen removal functional genes (*amoA*, *nirK*, *nirS*, *napA*, *narG* and *nosZ*) and the co-occurrence of various nitrifiers, denitrifiers, aerobic denitrifiers, and anammox bacteria. Aqeel and Liss (2020) found that in laboratory-scale bioreactors, i.e., sequencing batch reactor and a continuous stirred tank reactor, seeded with activated sludge from a municipal wastewater treatment plant and fed synthetic wastewater with no organics (SBR) (CSTR) and increasing concentrations of ammonia led to the enrichment of the nitrifying bacteria *Nitrosomonas*, *Nitrospira*, and *Nitrobacter* [16S rRNA gene sequencing (Illumina)] after 306 days. These autotrophic nitrifiers maintain heterotrophic organisms that grow on soluble microbial products released by the nitrifiers and on dead biomass (e.g., Ni et al. 2011).

More than nine years ago, a laboratory-scale continuous stirred tank reactor (CSTR) was filled with sludge from a wastewater treatment plant as inoculum (Ramírez-Vargas et al. 2013). Since then, NH_4_^+^ as energy source and sodium bicarbonate (NaHCO_3_) as the sole C source were added regularly to the reactor. As a result, a unique microbial community developed with autotrophic and heterotrophic microorganisms interacting with each other. After 5 years, the bacterial population was investigated a first time by 454 pyrosequencing the bacterial 16S rRNA gene (Ramírez-Vargas et al. 2015). Results showed a nitrifying community composed by bacteria belonging to *Nitrosomonas* (relative abundance 11.0%) as the sole AOB and *Nitrobacter* (9.3%) as the sole NOB. Pseudomonadota (62.8%) were the dominant bacterial phyla with Bacteroidota, known to metabolize extracellular polymeric substances produced by nitrifying bacteria and secondary metabolites of the decayed biomass, the second most abundant phylum (30.8%). The 16S rRNA gene of Archaea could not be amplified, although AOA would have contributed to the nitrification process as they do in most ecosystems (e.g. Haiming et al. 2022). Similarly, Fukushima et al. (2013) reported a prevalence of AOB such as *Nitrosomonas nitrosa* and *N. europaea*, while the genus *Nitrobacter* constituted the primary NOB population. While other studies have characterized both bacterial and archaeal communities, these typically involve systems with significantly shorter operational periods (e.g., González et al., 2022; Gu et al. 2019) or the use of different substrates, such as nitrite (e.g., Xiang et al. 2023).

Shotgun metagenomics, i.e., high-throughput sequencing and computational pipelines, allows to identify more microorganisms than amplification of the 16S rRNA gene and determine the genes found in the studied habitat. Therefore, the bacterial and archaeal community in a nitrifying reactor maintained under the same conditions for more than 9 years was determined using shotgun metagenomics so that a deeper insight into its composition and functionality was obtained. The reactor was sampled consecutively 7 times in September and October 2017 to determine temporal variability and three times at same moment on 26th of October to investigate variability within the reactor (Supplementary Table S1). It was hypothesized that the specific conditions in the reactor and the long-term experiment (nine years) would select for a specialized and limited archaeal and bacterial community that might help us understand the metabolic links between autotrophic and heterotrophic microorganisms. However, the nitrifiers maintained a highly diverse archaeal and bacterial community and the heterotrophic metabolic activity in the reactor was diverse and highly complex. This is the first time, as far as we are aware, that a nitrifying reactor was maintained under stable conditions for such a long time (> nine years) and that the bacterial and archaeal population was determined in such detail.

## Materials and methods

### Autotrophic mixed culture

Details of the glass CSTR can be found in Ramírez-Vargas et al. (2013). Briefly, the CSTR (0.14 m diameter, 0.56 m height and 5.5 L working volume) was inoculated on March 26, 2012, with 0.5 L of suspended biomass obtained from a previous nitrifying reactor started up in 2008 (Supplementary Fig. S1). The original reactor was inoculated with sludge from a full-scale aerobic nitrifying reactor treating domestic wastewater (Autonomous Metropolitan University, Mexico). Ammonium was added to the CSTR as energy source and bicarbonate as C source, so an autotrophic nitrifying population was selected. The CSTR was operated continuously under the same conditions for nine years to study the bacterial community, and kinetic and stoichiometric parameters through respirometric techniques. The reactor was fed with a solution containing (g/L distilled water); (NH_4_)_2_SO_4_, 1.73; NH_4_Cl, 1.40; KH_2_PO_4_, 2.73; FeCl_3_, 0.012; MgSO_4_, 0.60; NaCl, 1.00; CaCl_2_, 0.05; NaHCO_3_, 9.3 and 5.0 mL/L of a trace element solution containing (g/L): (NH_4_)_6_Mo_7_O_24_·4H_2_O, 0.08; ZnSO_4_·7H_2_O, 0.1; CuSO_4_·5H_2_O, 0.02; CoCl_2_·6H_2_O, 0.002; MnCl_2_·4H_2_O, 0.2. The solution did not contain organic C and only C in inorganic bicarbonate form was applied to the reactor. Ammonium and bicarbonate concentrations were such that a C/N ratio 1.8 was maintained, which was superior to the stoichiometric ratio of 0.85 required to achieve complete nitrification (Benninger and Sherrard 1978) while providing additional buffer capacity.

The mineral solution was fed with a constant flow rate of 0.69 L/d (hydraulic residence time of 8 days), using a peristaltic pump (Masterflex L/s precision, Cole-Parmer, USA). Air was supplied continuously at a flow rate of 1 vvm controlled by a mass flow controller (GFC171S, Aalborg, Denmark). The solids retention time (θc) for the CSTR was 55 days (Cervantes et al., 2006). The dissolved oxygen was maintained at > 5 mg O_2_/L and monitored with a polarographic dissolved oxygen bench meter (HI2400, Hanna Instruments). The pH was maintained at 7.5 ± 0.2 using either 1 M NaOH or 1 M H_3_PO_4_. The reactor was maintained at room temperature (21 ± 2 °C). During operation, effluent biomass was settled and part of it was recycled to the reactor to increase the biomass concentration. The ammonium influent and effluent, and nitrate and nitrite effluent concentrations were monitored from the onset of the operation of the reactor at regular intervals. Ammonium, nitrate, and nitrite were measured in triplicate according to standard colorimetric methods. Ammonium concentrations were expressed as nitrogen oxygen demand (NOD), defined as the theoretical mass of oxygen required to oxidize 1 g of NH_4_^+^ -N to nitrate (4.57 g NOD g^−1^ NO_3_^−^ -N). Nitrogen mass balance was confirmed through total nitrogen measurement (Shimadzu TOC-Vcsn equipped with a TNM-1 module, Shimadzu, Mexico) (Ramírez-Vargas et al. 2013). A more detailed discussion of the operation characteristics can be found in Ramírez-Vargas et al. (2013, 2015).

### Extraction of total DNA

Three 10 mL sub-samples were taken from the bioreactor with a week interval eight times and total DNA was extracted with enzymatic lysis using the automated QIAcube robot (Qiagen, Venlo, The Netherlands). Aliquots of 2 mL of each sample were centrifuged at 12,000 × *g* for 5 min and the pellets were resuspended in 200 *μ*L sterile distilled water. For lysis, 180 *μ*L lysozyme (20 mg/mL) were added to each tube, followed by incubation at 37 °C for 1 h. The samples were processed by the QIAcube-robot with the QIAamp DNA Mini (Qiagen) and eluted in a final volume of 200 *μ*L water.

The integrity of DNA was confirmed on 0.8% agarose gel and quantified using the Invitrogen’s PicroGreen® dsDNA fluorometric quantitation assay on a NanoDrop™ 3300 (Thermo Scientific, Carlsbad, CA). The DNA was sequenced by Macrogen Inc. (Seoul, Korea) using a HiSeq2000 Illumina® 2 × 100 paired-end platform. The raw sequences dataset was deposited in the NCBI-SRA (Sequence Read Archive) under BioProject accession number PRJNA770100 (https://www.ncbi.nlm.nih.gov/bioproject/PRJNA770100).

### Bioinformatics and assembly

First, raw paired-end reads were mapped against the masked human reference genome GRCh37/hg19 using BBMap (https://sourceforge.net/projects/bbmap/) to identify and remove possible human contaminant sequences as recommended by Bushnell et al. (2014). Unmapped reads to the human genome were quality filtered using Sickle v1.33 (Joshi et al., 2011), i.e., paired-end sequences with an average Q-score < 20 and shorter than 50 nucleotides (nt) were discarded. Unambiguous and unpaired reads that passed quality filtering were kept (-s, singles or uneven pair) for assembly.

High quality reads were used as inputs for the MEGAHIT assembler (Li et al. 2015). The multiple k-mer lengths option was chosen based on the software-developer’s recommendations for large and complex metagenomes (e.g., soil samples (–k-min 27 and –k-step 10)), and the minimum contig length was set to 200 nt.

### Gene prediction and functional annotation

Open reading frames prediction in assembled contigs was done with Prokka v1.13 using the metagenome setting for highly fragmented datasets (https://github.com/tseemann/prokka) (Seemann and Prokka, 2014), which were then subjected to a BLAST search (Altschul et al. 1990) and compared to the NCBI’s non-redundant protein database using DIAMOND (Buchfink et al. 2015) to achieve functional annotation (e-value cut-off ≤ 0.001). The BLAST results were analyzed with MEGAN CE v6.1 (Huson et al. 2016) and genes were assigned to functional categories, such as the evolutionary genealogy of genes (hee and after eggNOG) (subsystems Level 2 and Level 3) (Powell et al. 2012), functional roles based on SEED hierarchy (subsystems Level 1 and Level 2) (Overbeek et al. 2014), and InterPro families (InterPro2Go classifier) (Huson et al. 2016; Mitchell et al. 2015). The BLAST results analyses through MEGAN were done with the following settings: 60% as both min Score and Min Percent Identity, normalized counts, unassigned reads were ignored and the “Long Reads LCA” algorithm was used.

Metabolic pathways prediction was done as follows. The generic feature annotation file (GFF Prokka’s annotation output) was filtered to retain all genes with a KO identifier assigned. The "Minimal set of Pathways" tool (MinPath v1.4, (Ye and Doak 2009)) was used to obtain a conservative estimation of pathways to visually represent them on the microbial metabolic map through the interactive pathway explorer web-based tool (iPath v3, https://pathways.embl.de/).

### Nitrogen cycle related genes

Sequences were aligned against the NCyc database [68] using DIAMOND aligner to a 95% identity (Buchfink et al. 2015). The NCyc database was constructed with nitrogen cycle gene families from UniProt and from different orthology databases (COG, SEED, KEGG and eggNOG) (https://github.com/qichao1984/NCyc) (Tu et al. 2019).

### Microbial community analysis

Human cleaned metagenomic reads were analyzed with Kraken2 for the microbial community composition analysis using a RefSeq pre-built reference database constructed with bacterial and archaeal published complete genomes on 22nd of October 2019 (Wood et al. 2019). The results obtained with Kraken2 were analyzed using the Bayesian re-estimation of abundance with Bracken (Lu et al. 2017) using default parameters (Bracken “read length” was set to 100). Bracken’s taxonomy results were converted to a biological observation matrix (biom) with kraken-biom tool (https://github.com/smdabdoub/kraken-biom), and down-sampled with the minimum number of reads assigned for each database used.

The reactor archaeal and bacterial diversity was determined using the Hill numbers at different q orders (q = 0, 1 and 2) using the raw count dataset at the genus taxonomic level as described by Ma and Li (2018). Hill numbers have the advantage over commonly used diversity indices that they maintain the same measurement unit across values, i.e., effective number of species, overcoming the bias due to rare and dominant species and they are comparable with the traditional diversity indices.

### Statistical analysis

The statistical analysis was done in R (version 4.0.2, (R Core Team 2019)). Ordination (principal component analysis (PCA)) was done with converted sequence data using the centered log-ratio transformation test returned by the aldex.clr argument (ALDEx2 package version 1.21.1.) prior to PCA. The PCA was used to explore temporal and spatial variability, i.e., samples taken on the same day, of the bacterial groups in the reactor using FactoMineR package version 2.3.

## Results and discussion

Aerobic oxidation of NH_3_ to NO_2_^−^ and NO_2_^−^ to NO_3_^−^ by autotrophic nitrifiers generates energy for growth. No organic C was applied to the reactor so metabolites from the nitrifiers and their cell material provided organic material for heterotrophs. Surprisingly, that organic material sustained a wide range of bacteria and archaea even after nine years of applying only inorganic C (NaHCO_3_) to the reactor.

### Mineral N dynamics in the reactor

**Fig. 1:**
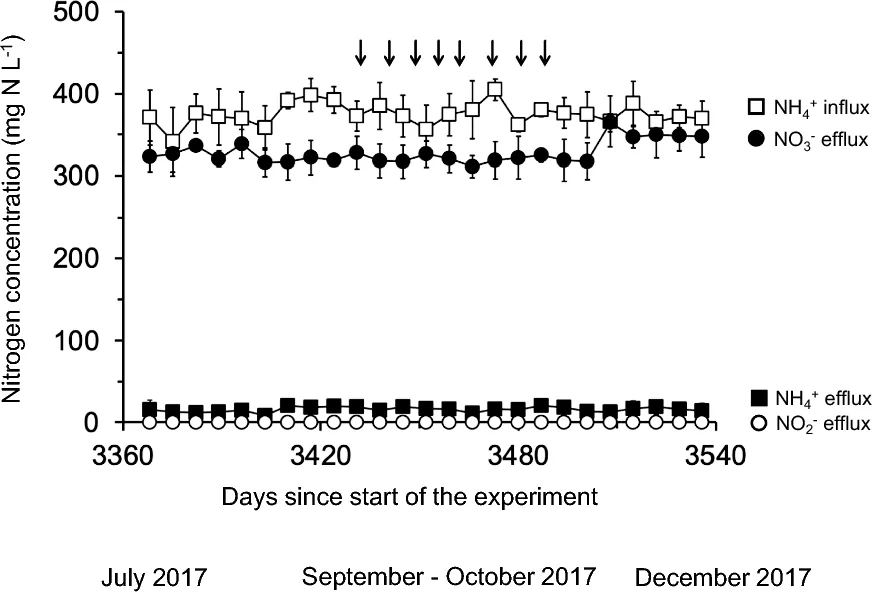
Nitrifying reactor with NH_4_^+^ influent concentration, NH_4_^+^ effluent concentration, NO_3_^−^ effluent concentration and NO_2_^−^ effluent concentration. Arrows indicate DNA sampling dates

The NH_4_^+^-N content in the influent was on average 375 mg NH_4_^+^-N L^−1^ while that of the effluent ranged between 8.7 and 20.5 mg NH_4_^+^-N L^−1^ with an average of 15.8 mg NH_4_^+^-N L^−1^ (Fig. 1). The NO_2_^−^-N content in the effluent was on average 0.11 mg The NO_2_^−^-N L^−1^ while the NO_3_^−^-N content ranged between 311 and 350 mg NO_3_^−^-N L^−1^ with an average of 328 mg NO_3_^−^-N L^−1^. The ammonia removal efficiency, i.e., the percentage of ammonia that was oxidized, ranged 94.5–97.7% with an average of 95.8%. Of these, 91.3%, on average, were converted into nitrate, and 0.03% into nitrite. The nitrifier reactor was working well and under steady state after nine years of continuous operation. Most of the NH_4_^+^ in the influent was oxidized and the removal rate was 95.8 ± 3.1% during sampling period. Consequently, the concentration of NO_2_^−^ remained low (< 0.2 NO_2_^−^-N L^−1^) and most of the NH_4_^+^- added to the reactor was converted to nitrate, with an average of 91.3%. This suggested a well-developed nitrifier population with high and uninhibited activity.

### Bacterial community structure

**Fig. 2:**
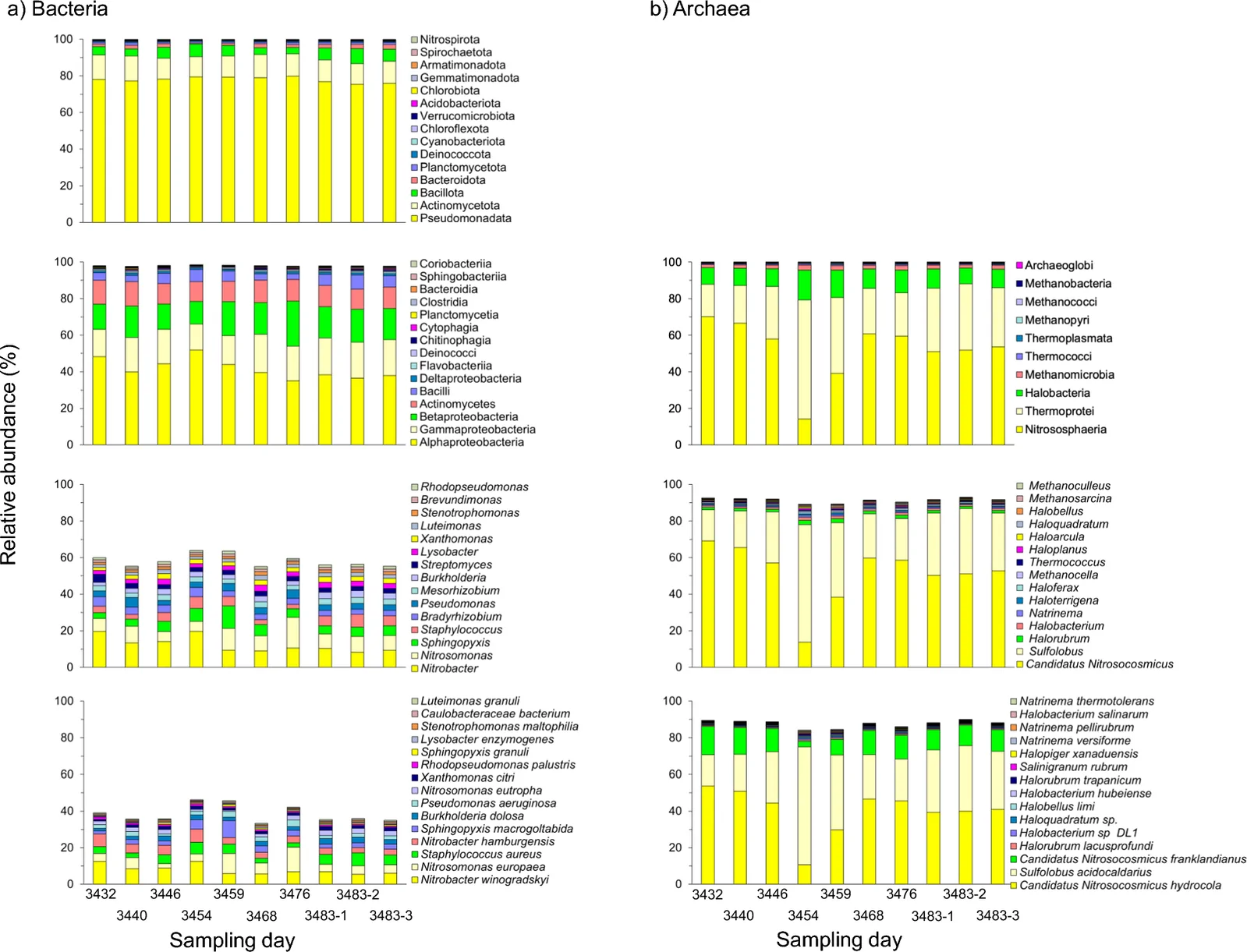
Relative abundance (%) of **a** bacterial and **b** archaeal phyla, classes, genera and species in the continuous stirred tank nitrifying reactor

Overall, 70,606,106 bacterial sequences were obtained from the CSTR reactor that included 38 different bacterial phyla, 1242 genera and 4483 species (Supplementary Table S2). The metagenome alpha diversity (qDα) based on Hill equivalent numbers showed a larger variation over time at q = 1 (385 ± 101 (mean ± standard deviation of the samples taken at separate times) and q = 2 (50 ± 18) than at q = 0 (4222 ± 180) (Supplementary Fig. S2). All q values also showed spatial variation (q = 0: 3979 ± 202, q = 1: 483 ± 54, q = 2: 70 ± 14). The Pseudomonadota (relative abundance 78.44 ± 1.28%, mostly Alphaproteobacteria (42.58 ± 5.59%)) and Actinomycetota (12.12 ± 0.93%) were the dominant bacterial phyla (Fig. 2a). Members of *Nitrobacter* (13.12 ± 4.47%), i.e., oxidizers of nitrite, and *Nitrosomonas* (9.07 ± 3.77%), i.e., oxidizers of ammonium dominated the bacterial genera. The relative abundance of *Sphinopyxis* (5.96 ± 2.78%) and *Staphylococcus aureus* (4.21 ± 1.60%) was also high. Bacterial NH_3_ oxidizers detected in the reactor included six species of *Nitrosomonas* and three members of *Nitrosospira*. *Nitrosomonas europaea*, the most abundant bacterial NH_3_ oxidizer in the reactor, is considered an obligate chemolithoautotroph although it can use limited amounts of some organic compounds, such as fructose (Hommes et al. 2003). The relative abundance of *Nitrosospira* (0.24%), a known contributor to N_2_O emissions when NH_3_ is oxidized, was much lower in the reactor than that of *Nitrosomonas* (9.07%) (Lourenço et al. 2018). Members of *Nitrosomonas* often dominate in a denitrification-nitrification bioreactor although phylotypes belonging to *Nitrosospira* have been found to dominate in a nitrifying reactor at pH 6.0 (Wang et al. 2016).

Two NOB genera were detected in the reactor, i.e., *Nitrobacter* represented by two and *Nitrospira* by three species, although the relative abundance of the first was much higher than that of the latter. There are two reasons, i.e., the dissolved oxygen and/or the concentration of NO_2_^−^, which might explain why the relative abundance of members of *Nitrospira* was much lower than that of *Nitrobacter* (Huang et al. 2010). First, phylotypes belonging to *Nitrospira* have a higher affinity for oxygen and nitrite so they are enriched in low-oxygen environments over *Nitrobacter* spp. (Downing and Nerenberg 2008). The oxygen supply in the reactor studied was high, so phylotypes belonging to *Nitrobacter* were enriched. Second, *Nitrobacter* is an r-strategist with rapid growth and dominates when concentrations of NO_2_^−^ are high, while *Nitrospira*, i.e., a *K*-strategist that grows more slowly, dominates when resources are limited (Nogueira and Melo 2006). As such, *Nitrobacter* prefers environments with higher concentrations of NO_2_^−^ while *Nitrospira* is enriched when NO_2_^−^ concentrations are low (Bartosch et al. 1999). Copious amounts of NH_4_^+^ were added to the reactor so phylotypes belonging to *Nitrobacter* were enriched as oxidation of NH_4_^+^ will provide NO_2_^−^ that serves as energy source for them. The long-term microbial composition observed in this study reflects a remarkable functional and taxonomic stability within the bioreactor. When comparing our results with the characterization performed at the five-year mark (Ramírez-Vargas et al. 2015), the core nitrifying community —predominantly composed of the ammonia-oxidizing bacterium (AOB) *Nitrosomonas europaea* and the nitrite-oxidizing bacterium (NOB) *Nitrobacter winogradskyi*— has remained consistently dominant. Despite the technical differences in sequencing depth and resolution between 454-pyrosequencing and shotgun metagenomics and the persistence of these key taxa under identical operational parameters (Ramírez-Vargas et al. 2013) suggests that the system has reached a ‘climax community’.

Phylotypes belonging to *Candidatus* Nitrospira inopinata were detected in the bioreactor and although their relative abundance was low (2.2 × 10^–3^%), they consisted nearly 10% of the genus *Nitrospira*. *Candidatus* Nitrospira inopinata was described as a complete ammonia oxidizer (Comammox) (Daims et al. 2015). It was isolated from a microbial biofilm developing on the walls of a pipe under the flow of hot water (56 °C, pH 7.5) raised from a 1200 m deep oil exploration well (Aushiger, North Caucus, Russia) and incubated at 46 °C in ammonium-containing mineral medium to enrich moderately thermophilic ammonium oxidizing microorganisms. Xia et al. (2018) using a two-step PCR using highly degenerate primers (THDP-PCR) and quantitative real-time PCR (qPCR) stated that “The high proportions of complete ammonia oxidizers (comammox) in various environments, based on the qPCR and public metagenomic data set analyses, support the ubiquitous distribution”. Spasov et al. (2020) using quantitative PCR (qPCR), 16S rRNA gene sequencing, and metagenomics, reported that comammox *Nitrospira* were more abundant than all other nitrifiers in biofilm samples collected from tertiary rotating biological contactors (RBCs) of a municipal wastewater treatment plant in Canada and they might dominate in low ammonia environments (Al-Ajeel et al. 2022). Not only autotrophic bacteria were detected in the reactor but also heterotrophic nitrifiers, such as *Pseudomonas tolaasii* (He et al. 2016) and heterotrophic aerobic denitrifiers, such as *Klebsiella variicola* and *K. pneumoniae* (Feng et al. 2018). Both bacterial groups have been successfully combined to remove COD (84.13%) and total N (91.54%) in a membrane aerated biofilm reactor by inoculating conventional activated sludge (Lan et al. 2022). Although the nitrifying reactor was well oxygenated, it has to be assumed that some microenvironments, i.e., flocs were low in O_2_ content. The bacteria *Candidatus* Kuenenia stuttgartiensis capable of anammox (relative abundance 0.76 × 10^–3^%), was detected confirmed by the presence of the hydrazine oxidoreductase encoding (*hzo)* gene (Strous et al. 2006).

### Archaeal community structure

**Fig. 3:**
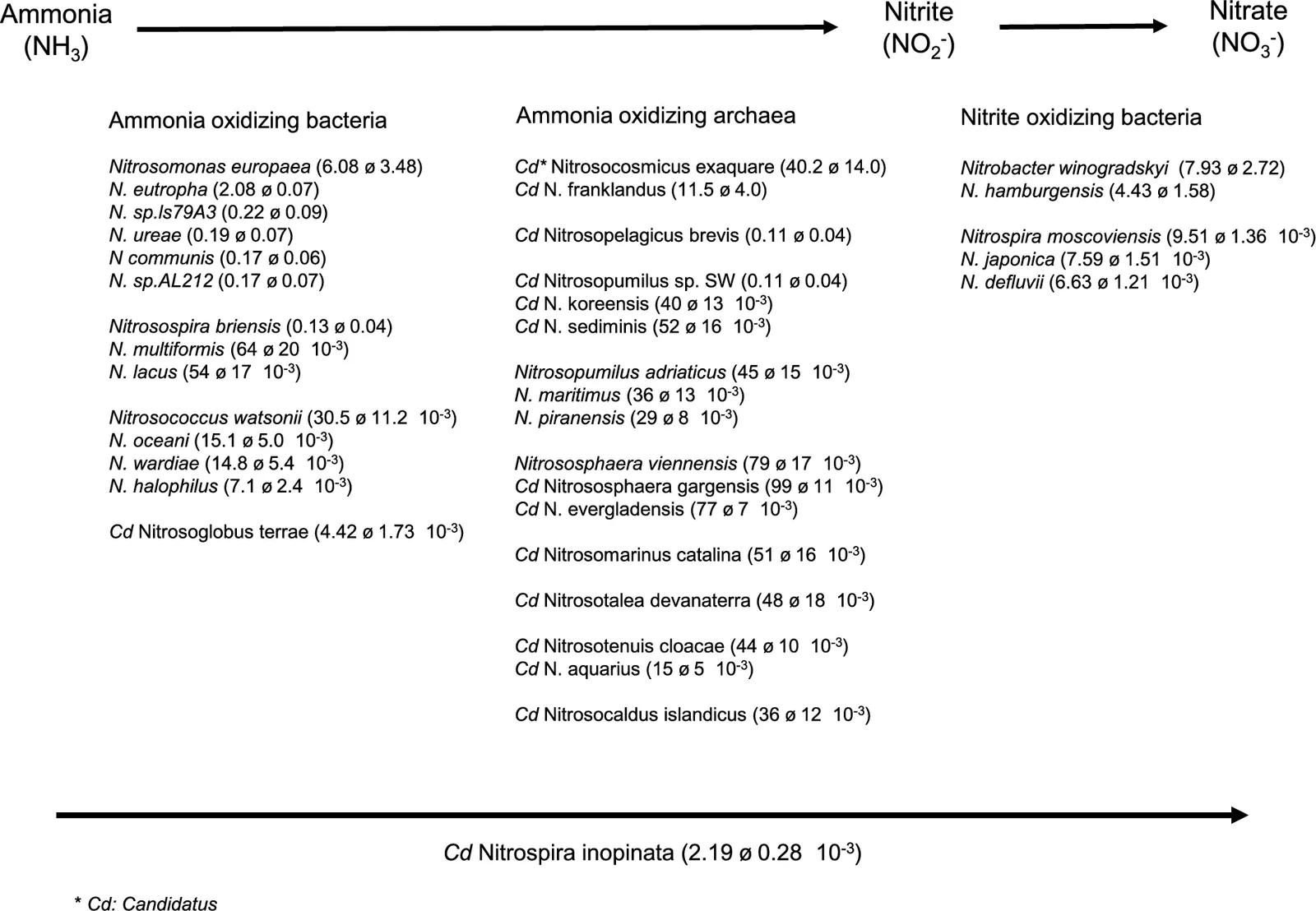
Bacterial and archaeal groups involved in the nitrification process found in the continuous stirred tank nitrifying reactor. Values between parenthesis are relative abundance ± standard deviation

Overall, 8,071,654 archaeal sequences were extracted from the CSTR reactor (Supplementary Table S1). The CSTR reactor sustained also a rich archaeal community with 5 different phyla, 110 genera and 245 species. Nitrososphaerota (52.56 ± 18.16%, mostly Nitrososphaeria (52.56 ± 18.16%)) and Thermoproteota (32.05 ± 15.42% mostly Thermoprotei (32.05 ± 15.42%)) were the dominant archaeal phyla (Fig. 2b). Ammonium oxidizing archaea mostly *Candidatus* Nitrosocosmicus hydrocola (40.22 ± 13.97%) and *Candidatus* N. franklandianus (11.47 ± 3.99%) dominated in the CSTR. Sulfolobus acidocaldarius (31.37 ± 15.39%) was the second most dominant archaeal species in the CSTR reactor. The archaeal community was dominated by three species, i.e., *Candidatus* Nitrosocosmicus hydrocola and *Ca*. N. franklandianus, and *Sulfolobus acidocaldarius*. Their relative abundance was > 10%, while that of the remaining archaeal species was < 0.6%. *Candidatus* Nitrosocosmicus hydrocola and *Ca*. N. franklandianus belong to the Nitrososphaeria (Nitrososphaerota). They are chemolithoautotrophs that depend on the oxidation of ammonia for growth (Kerou et al. 2016). Ammonia oxidizing archaea are generally adapted to environments with low ammonia contents, e.g., treatment of drinking water treatment, tertiary wastewater treatment systems, and aquaculture/aquarium filtration while high concentrations normally favour AOB (Al-Ajeel et al. 2022). However, the addition of a constant high amount of NH_4_^+^ enriched two AOA archaea adapted to high NH_3_ concentrations. *Candidatus* N. hydrocola, the most abundant archaeal species in the reactor studied, was isolated from an industrial and municipal wastewater treatment plant and tolerated at least 15 mM of ammonium chloride or sodium nitrite (Sauder et al. 2017). *Candidatus* N. franklandianus, isolated from soil, is also tolerant to high concentrations of ammonia (Lehtovirta-Morley et al. 2016). Fifteen other members of the Nitrososphaeria were detected in the reactor, but their relative abundance was ≤ 0.11% (Fig. 3).

In the anammox process, ammonium is oxidized to dinitrogen gas using nitrite as the electron acceptor under anoxic conditions. Members of seven methanogenic orders, i.e., Methanobacteriales, Methanocellales Methanococcales, Methanomassiliicoccales Methanomicrobiales, Methanopyrales and Methanosarcinales belonging to the phylum Methanobacteriota were detected in the reactor (Enzmann et al. 2018). Methanogens are strict anaerobes and do not grow when O_2_ is available (Lyu et al. 2018). Species of the four classified genera of the strict anaerobe Methanobacteriaceae (relative abundance 0.18%) were detected in the reactor. During hydrogenotrophic methanogenesis, H_2_ is oxidized to H^+^, and CO_2_ is reduced to CH_4_, but species of Methanosphaera, i.e., *Methanosphaera* sp. BMS and *M. stadtmanae*, do not reduce CO_2_ and obtain their energy only from the reduction of methanol by H_2_ (Oren 2014). Moreover, other methanogens from the orders Methanosarcinales and Methanomassiliicoccales, e.g., *Methanimicrococcus blatticola* and *M. luminyensis*, have a similar use of methanol (Feldewert et al. 2020; Mand and Metcalf 2019).

### Genes involved in nitrogen cycling

**Fig. 4:**
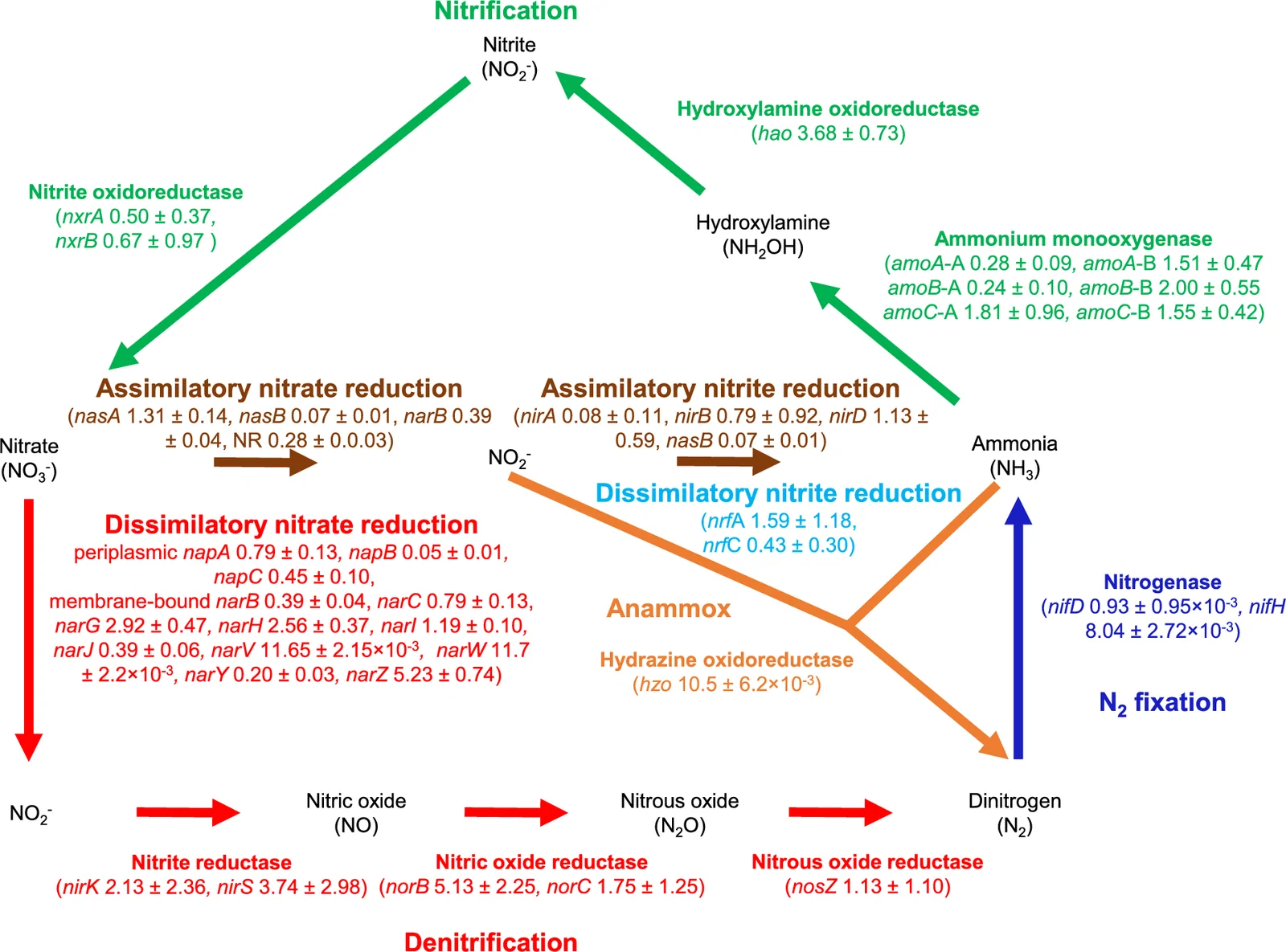
Genes involved in the most important N cycling processes. Values between parenthesis are relative abundance ± standard deviation

Sequences of a wide range of genes related to different processes involved in the N cycle, i.e., nitrification, denitrification, N_2_ fixation, anammox and assimilatory NO_3_^−^ reduction, were detected in the reactor (Fig. 4). Genes for six ammonium monooxygenases, i.e., three from bacteria and three from archaea, were detected in the reactor and a wide range of genes that code for enzymes involved in assimilatory and dissimilatory nitrate reduction. Genes that code for nitrite, nitric oxide and nitrous oxide reductases were found, but interestingly also hydrazine oxidoreductase (*hzo* gene), which is involved in the anammox process, moreover a wide range of genes related to the most important processes involved in N cycling were detected in the nitrifying reactor (Supplementary Table S3, Fig. 4).

Three different enzymes participate in the oxidation of NH_3_ to NO_3_^−^, ammonium monooxygenase, hydroxylamine oxidoreductase and nitrite oxidoreductase, encoded often by different genes. The amo operon in ammonium oxidizing bacteria and archaea consists of at least three genes *amoA*, *amoB* and *amoC* with *amoA* encoding for the subunit containing the putative enzyme active site (Norton et al. 2002; You et al. 2009). All six genes were detected in the reactor. Hydroxylamine oxidoreductase oxidizes hydroxylamine (NH_2_OH) to NO_2_^−^ and the hydroxylamine oxidoreductase gene cluster (*hao*) is highly conserved in autotrophic NH_3_^−^ oxidizing bacteria, such as *Nitrosococcus oceani*, *Nitrosospira multiformis* and *Nitrosomonas europaea* (Bergmann et al. 2005). Nitrite oxidoreductase is the key enzyme responsible for the oxidation of NO_2_^−^ to NO_3_^−^ in nitrite-oxidizing *Nitrobacter* strains and the *nxrB* gene is a functional and phylogenetic marker for species belonging to *Nitrospira* (Pester et al. 2014).

A wide range of genes that coded for enzymes involved in assimilatory and dissimilatory nitrate reduction were detected in the reactor studied. Nitrate is reduced to provide N (assimilatory nitrate reduction), as terminal electron acceptor or to dissipate excess energy (dissimilatory nitrate reduction) (Feng et al. 2014). Assimilatory nitrate reductase is encoded by *nas* genes (*nasA* and *nasB* in the nitrifying reactor), but also by the membrane bound *narB* gene (Feng et al. 2014; Hidalgo-García et al. 2019). Dissimilatory nitrate reductases are encoded by a wide range of genes. Some dissimilatory nitrate reductases are periplasmic and encoded by the *napA*, *napB* and *napC* genes, while others are membrane bound and encoded by the *narG*, *narH*, *narI* and *narJ* genes (Cole 1996; Heylen and Keltjens 2012).

Several genes that coded for enzymes involved in assimilatory, i.e., *nirA*, *nirB* and *nirD*, and dissimilatory nitrite reduction, i.e., *nrfA* and *nrfC*, were detected in the reactor studied. The formed NO_2_^−^ can be reduced further by dissimilatory nitrite reductases to NH_3_ encoded by the *nrfA* and *nrfC* genes and assimilatory NO_2_^−^ reductase encoded by the *nirA* and *nirB* genes. Nitrite can also be reduced to nitric oxide by nitrite reductase (denitrification) encoded by the *nirK* and *nirS* genes.

Well known genes that coded for enzymes involved in the denitrification process, i.e., *nirK* and *nirS*, *norB* and *norC*, and *nosZ*) were detected in the reactor studied. Denitrification is the reduction of NO_3_^−^ to N_2_O or N_2_ and approximately 50 genes are required in a bacterium to encode the core structures of the denitrification apparatus (Zumft 1997). Denitrification in Gram-negative bacteria occurs mostly in the periplasm, but that of Gram-positive bacteria is not as well ascertained (Zumft 1997). The reduction of NO_2_^−^ to nitric oxide (NO) is catalyzed by two biochemically and genetically different nitrite reductases, the copper containing NirK and cytochrome *cd1* protein (NirS) (Bushnell et al., 2014). Nitric oxide is further reduced to nitrous oxide by nitric oxide reductases which are membrane proteins in bacteria and are classified as cytochrome *cb* heterodimers (Hendriks et al. 2000).

The capacity to fix N_2_ is found in different bacterial and archaeal groups catalyzed mostly by the molybdenum nitrogenase (Li et al. 2014). Only two genes that encoded for enzymes involved in N_2_ fixation process, i.e., *nifD* and *nifH*, were detected in the reactor studied. More in-depth studies, e.g., with ^15^N, might be required to investigate if N_2_ fixation actually takes place in the reactor and how much N_2_ was fixed as the reactor was supplied with a large amount of NH_4_^+^, which normally inhibits N_2_ fixation.

The high abundance of denitrification-related genes, particularly nitrite reductase (*nirS* 3.74 ± 2.98) and nitric oxide reductase (*norB* 5.13 ± 2.25), reveals a robust potential for nitrogen loss within the same aerobic environment. The prevalence of *nirS* over *nirK* (2.13 ± 2.36) is a common feature in stable wastewater treatment communities, indicating a specialized niche for nirS-type denitrifiers.

This coexistence of high nitrification (*hao*) and denitrification (*norB*) potential suggests the occurrence of simultaneous nitrification and denitrifaction (SND), likely facilitated by the micro-anaerobic zones within the flocculent biomass and the aerobic zone in reactor provided by the continuous stirring (Satoh et al. 2003). Interestingly, the extremely low abundance of the hydrazine oxidoreductase (*hzo*) gene (10.5 ± 6.2x^10⁻3^) and nitrogenase (*nifH*) genes confirms that Anammox and nitrogen fixation are negligible pathways in this reactor’s nitrogen balance. Instead, the nitrogen cycle is driven by a highly efficient ‘Nitrification–Denitrification’ axis, supported by the strong presence of membrane-bound nitrate reductases, which ensures the rapid turnover of nitrogenous species.

### Impact of database selection on bioinformatics analysis

The classification sensitivity and specificity of Kraken2 are fundamentally linked to the comprehensiveness of the reference database used. This dependency arises primarily from the vast number of environmental microorganisms that remain unsequenced, consequently, the lack of representative genomes can introduce significant taxonomic biases, thereby reducing classification performance at the species level (Lu et al. 2022). Despite these inherent constraints, Kraken2 remains a pivotal tool that, when integrated with high-throughput shotgun metagenomics, facilitates the exploration of complex microbial ecosystems and their functional potential (Lugli et al. 2019).

The most abundant functionality was dependent on the database used. The Eggnog database at level 2 indicated that genes related to amino acid transport and metabolism, and energy production and conversion were the most abundant functions (Supplementary Fig. S3a), while the SEED data base at level 2 indicated that genes related to catalytic activity and metabolic processes were the most abundant (Supplementary Fig. S3b online). The Interpro database at level 2 indicated that genes related to carbohydrate, and amino acids and derivate related metabolic activity were the most abundant (Supplementary Fig. S3c).

### Temporal and spatial variability

The difference in the bacterial community structure when samples were taken on the same day was smaller than the changes over time (Supplementary Table S4). The coefficient of variance of the temporal and spatial variation considering the mean of the different taxonomic levels was in the order bacteria > archaea. The relative abundance of the bacterial phyla showed slight variation over time, although some variation was found at the level of species, e.g., *N. winogradskyi*.

**Fig. 5:**
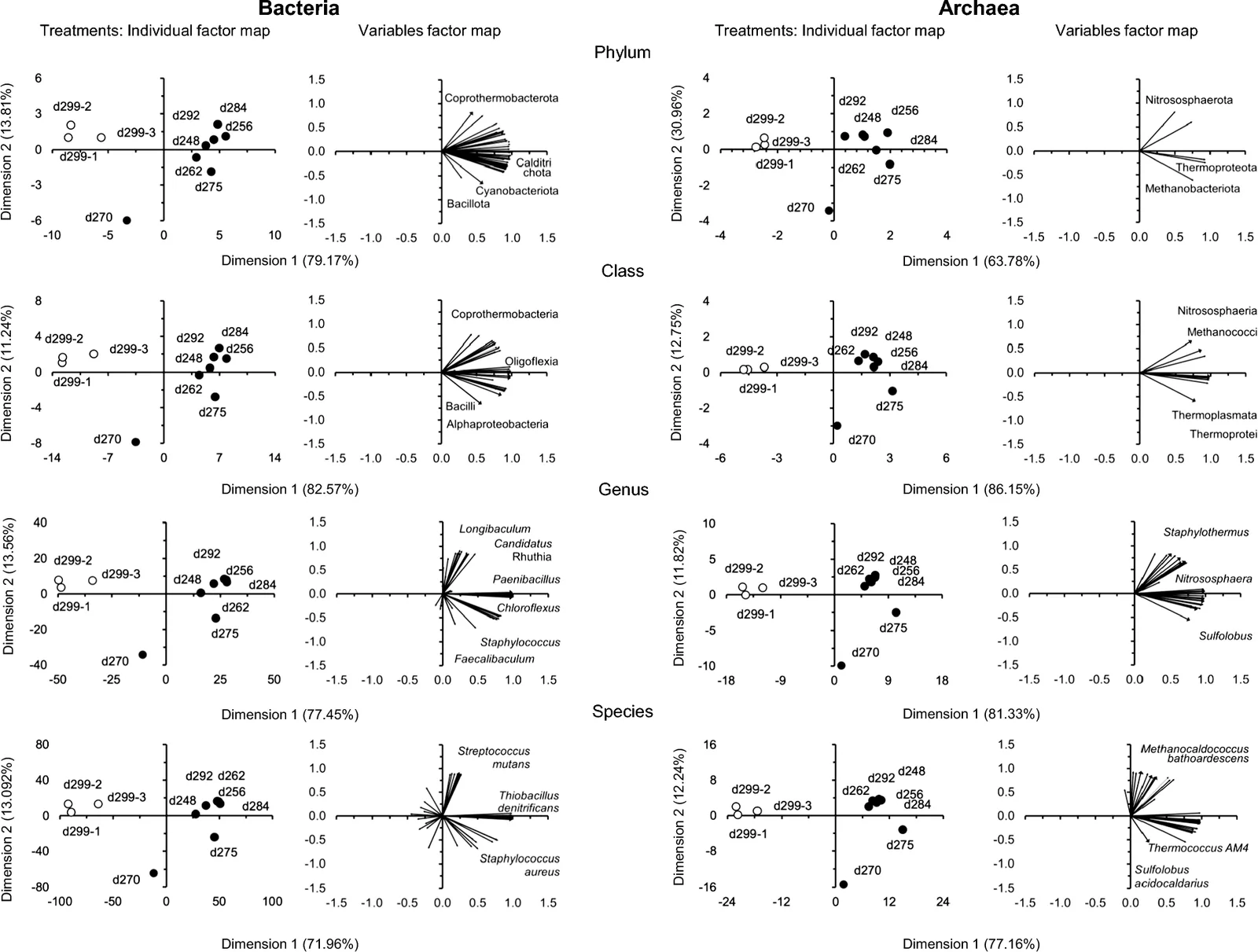
Principal component analysis (PCA) for variables and treatments in bacterial and archaeal communities

The PCA results for both bacteria and archaea (Fig. 5) showed that most of the variance was explained by the first dimension (PC1), accounting for 79.17% and 63.78%, respectively. A distinct clustering of temporal samples from day 248 to day 292 (d248, d256, d262, d284, and d292) was detected along the PC1 axis. This tight grouping suggests that the bioreactor had reached a climax community state, characterized by high taxonomic stability over time. This observation is consistent with the long-term (9-year) operation under constant parameters, which favors the maintenance of a core microbiome (Ramírez-Vargas et al. 2015). Sample d270 exhibits a slight shift along PC2, indicating a transient fluctuation in the community before returning to the established steady state.

To address the potential for spatial niche differentiation, replicates from the spatial series (d299-1, d299-2, and d299-3) were analyzed. These samples formed a cohesive cluster on the left quadrant of the PCA across all taxonomic levels, i.e., from phylum to species. The minimal distance between these spatial replicates proves a high degree of spatial homogeneity within the reactor. This uniformity is likely facilitated by the flocculent nature of the biomass and efficient hydraulic mixing, which prevents the formation of significant micro-environmental gradients that would otherwise promote spatial divergence in the microbial population (Ju and Zhang., 2015).

The variable factor maps identify the specific taxa responsible for the observed distribution. For bacteria, the community structure was primarily affected by members of the Coprothermobacterota, Bacillota, and Chloroflexus. The presence of *Thiobacillus denitrificans* as a driving species highlights the role of sulfur-cycling and denitrifying groups in the systems’ metabolic network. For Archaea, the dominance of Nitrososphaerota and the class Nitrososphaeria—which includes the genus Nitrososphaera—along the primary axis underscores the fundamental role of ammonia-oxidizing archaea (AOA) in maintaining the autotrophic nitrifying function of the reactor.

## Conclusions

After nine years of applying ammonium as energy source and inorganic C as only C source for aerobic autotrophic nitrifiers, a continuous stirred tank reactor originally inoculated with wastewater contained a highly diverse bacterial and archaeal community. Five different archaeal phyla, 110 genera and 245 species were detected and 38 bacterial phyla, 1242 genera and 4483 species. Four of these bacterial genera were ammonia oxidizers including 13 species and eight archaeal genera with 17 species. Only two bacterial nitrite oxidizing genera were found including five species. Interestingly, members of *Cd* Nitrospira inoinata that are capable of oxidizing NH_3_ to NO_3_^−^ and *Candidatus* Kuenenia-stuttgartiensis capable of anammox confirmed by the presence of the hydrazine oxidoreductase encoding (*hzo*) gene were detected. Although the nitrifying reactor was well oxygenated, it has to be assumed that some microenvironments existed, i.e., flocs that were low in O_2_ content, as not only *Candidatus* Kuenenia-stuttgartiensis, but also members belonging to seven different methanogenic strict anaerobic archaeal taxonomic orders were detected in the reactor.

Shotgun metagenomics allowed us to determine the autotrophic-heterotrophic archaeal-bacterial populations that structured the microbial community in an engineered environment, i.e., a nitrifying bioreactor after nine years. Future research should focus on FISH tools, high-throughput sequencing, MAG analysis and robust statistical analysis to provide a novel insight into populations in nitrifying reactors.

## Supplementary Information

Below is the link to the electronic supplementary material.Supplementary file1 (PPTX 56 KB)Supplementary file2 (PPTX 12143 KB)Supplementary file3 (PPTX 13861 KB)Supplementary file4 (DOCX 15 KB)Supplementary file5 (DOCX 18 KB)Supplementary file6 (DOCX 32 KB)Supplementary file7 (DOCX 19 KB)

## Data Availability

The author confirms that all data generated or analyzed during this study are included in this published article.
