# Supplementary material for: A nitrification bioreactor applied solely with ammonium and inorganic C maintains a highly diverse bacterial and archaeal community even after nine years
Source: Biodegradation. 2026 Jul 6;37(4):111. doi: 10.1007/s10532-026-10288-9 (PMC13337855; doi:10.1007/s10532-026-10288-9)
Supplement: Supplementary file 1 — Supplementary file1 (PPTX 56 KB) [file 10532_2026_10288_MOESM1_ESM.pptx]

## Slide 1
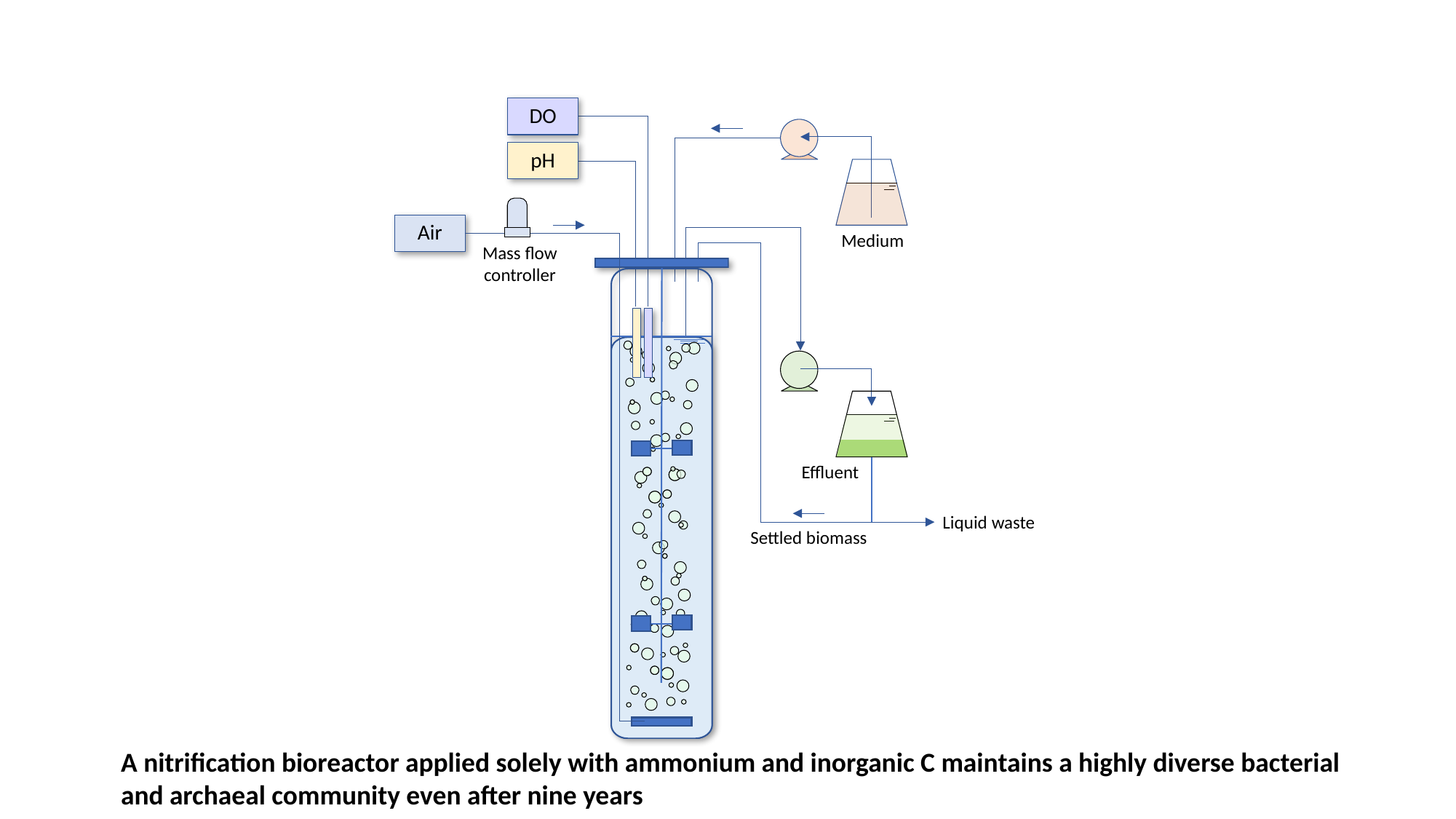

Medium
Mass flow controller
Effluent
Liquid waste
Settled biomass
A nitrification bioreactor applied solely with ammonium and inorganic C maintains a highly diverse bacterial and archaeal community even after nine years
