# Supplementary material for: A nitrification bioreactor applied solely with ammonium and inorganic C maintains a highly diverse bacterial and archaeal community even after nine years
Source: Biodegradation. 2026 Jul 6;37(4):111. doi: 10.1007/s10532-026-10288-9 (PMC13337855; doi:10.1007/s10532-026-10288-9)
Supplement: Supplementary file 2 — Supplementary file2 (PPTX 12143 KB) [file 10532_2026_10288_MOESM2_ESM.pptx]

## Slide 1
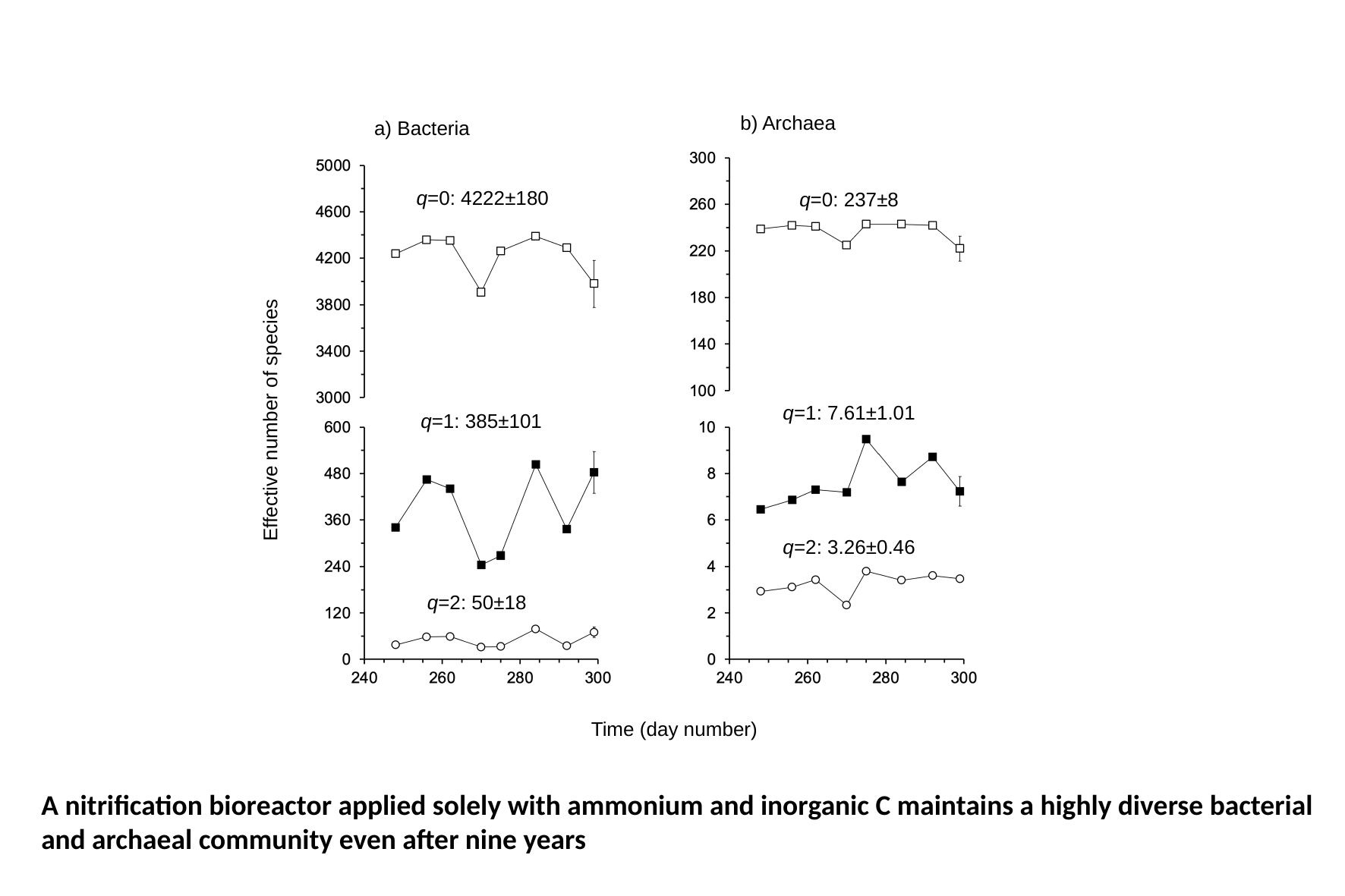

b) Archaea
a) Bacteria
q=0: 4222±180
q=0: 237±8
q=1: 7.61±1.01
Effective number of species
q=1: 385±101
q=2: 3.26±0.46
q=2: 50±18
Time (day number)
A nitrification bioreactor applied solely with ammonium and inorganic C maintains a highly diverse bacterial and archaeal community even after nine years
