# Supplementary material for: A nitrification bioreactor applied solely with ammonium and inorganic C maintains a highly diverse bacterial and archaeal community even after nine years
Source: Biodegradation. 2026 Jul 6;37(4):111. doi: 10.1007/s10532-026-10288-9 (PMC13337855; doi:10.1007/s10532-026-10288-9)
Supplement: Supplementary file 3 — Supplementary file3 (PPTX 13861 KB) [file 10532_2026_10288_MOESM3_ESM.pptx]

## Slide 1
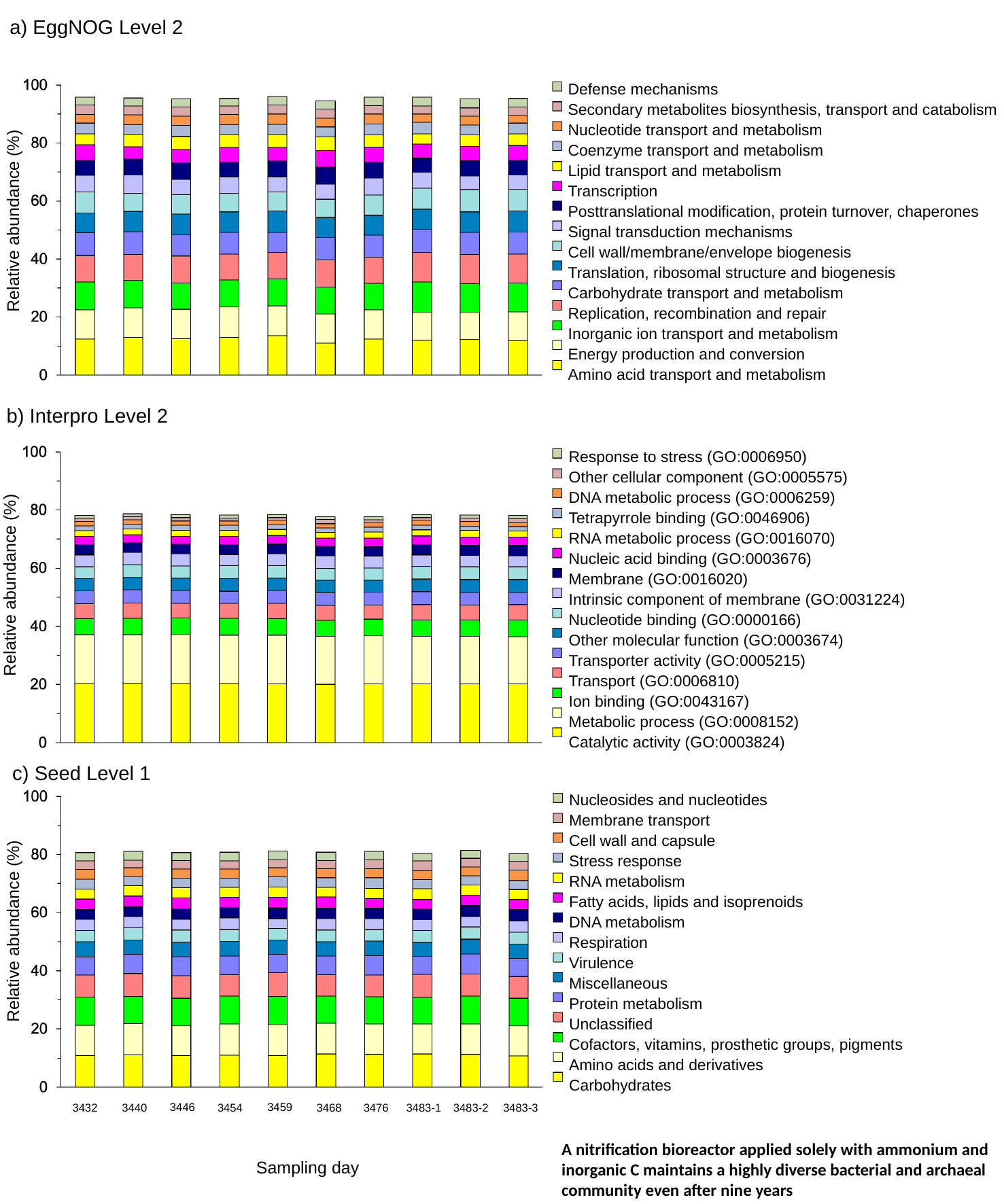

a) EggNOG Level 2
Defense mechanisms
Secondary metabolites biosynthesis, transport and catabolism
Nucleotide transport and metabolism
Coenzyme transport and metabolism
Lipid transport and metabolism
Transcription
Posttranslational modification, protein turnover, chaperones
Signal transduction mechanisms
Cell wall/membrane/envelope biogenesis
Translation, ribosomal structure and biogenesis
Carbohydrate transport and metabolism
Replication, recombination and repair
Inorganic ion transport and metabolism
Energy production and conversion
Amino acid transport and metabolism
Relative abundance (%)
b) Interpro Level 2
Response to stress (GO:0006950)
Other cellular component (GO:0005575)
DNA metabolic process (GO:0006259)
Tetrapyrrole binding (GO:0046906)
RNA metabolic process (GO:0016070)
Nucleic acid binding (GO:0003676)
Membrane (GO:0016020)
Intrinsic component of membrane (GO:0031224)
Nucleotide binding (GO:0000166)
Other molecular function (GO:0003674)
Transporter activity (GO:0005215)
Transport (GO:0006810)
Ion binding (GO:0043167)
Metabolic process (GO:0008152)
Catalytic activity (GO:0003824)
Relative abundance (%)
c) Seed Level 1
Nucleosides and nucleotides
Membrane transport
Cell wall and capsule
Stress response
RNA metabolism
Fatty acids, lipids and isoprenoids
DNA metabolism
Respiration
Virulence
Miscellaneous
Protein metabolism
Unclassified
Cofactors, vitamins, prosthetic groups, pigments
Amino acids and derivatives
Carbohydrates
Relative abundance (%)
3446
3459
3468
3483-1
3454
3432
3483-2
3476
3440
3483-3
A nitrification bioreactor applied solely with ammonium and inorganic C maintains a highly diverse bacterial and archaeal community even after nine years
Sampling day
