# Supplementary material for: A nitrification bioreactor applied solely with ammonium and inorganic C maintains a highly diverse bacterial and archaeal community even after nine years
Source: Biodegradation. 2026 Jul 6;37(4):111. doi: 10.1007/s10532-026-10288-9 (PMC13337855; doi:10.1007/s10532-026-10288-9)
Supplement: Supplementary file 4 — Supplementary file4 (DOCX 15 KB) [file 10532_2026_10288_MOESM4_ESM.docx]

Table S1. Detailed sampling for metagenomic analysis.

| Series | Sample ID | Date (DD/MM/YYYY) | Day of the Year | Operational Day |
| --- | --- | --- | --- | --- |
| Temporal | d248 | 05/09/2017 | 248 | 3432 |
|  | d256 | 13/09/2017 | 256 | 3440 |
|  | d262 | 19/09/2017 | 262 | 3446 |
|  | d270 | 27/09/2017 | 270 | 3454 |
|  | d275 | 02/10/2017 | 275 | 3459 |
|  | d284 | 11/10/2017 | 284 | 3468 |
|  | d292 | 19/10/2017 | 292 | 3476 |
| Spatial | d299-1 | 26/10/2017 | 299 | 3483 |
|  | d299-2 | 26/10/2017 | 299 | 3483 |
|  | d299-3 | 26/10/2017 | 299 | 3483 |
