# Supplementary material for: A nitrification bioreactor applied solely with ammonium and inorganic C maintains a highly diverse bacterial and archaeal community even after nine years
Source: Biodegradation. 2026 Jul 6;37(4):111. doi: 10.1007/s10532-026-10288-9 (PMC13337855; doi:10.1007/s10532-026-10288-9)
Supplement: Supplementary file 5 — Supplementary file5 (DOCX 18 KB) [file 10532_2026_10288_MOESM5_ESM.docx]

Table S2. The number of sequences, and bacterial and archaeal phyla, classes, orders, families, genera and species in the nitrifying laboratory-scale continuous stirred tank after nine years of similar conditions.

| ⎯⎯⎯⎯⎯⎯⎯⎯⎯⎯⎯⎯⎯⎯⎯⎯⎯⎯⎯⎯⎯⎯⎯⎯⎯⎯⎯⎯⎯⎯⎯⎯⎯⎯⎯⎯ | | | |
| --- | --- | --- | --- |
| Taxonomic level | Bacteria | Archaea |  |
| ⎯⎯⎯⎯⎯⎯⎯⎯⎯⎯⎯⎯⎯⎯⎯⎯⎯⎯⎯⎯⎯⎯⎯⎯⎯⎯⎯⎯⎯⎯⎯⎯⎯⎯⎯⎯ | | | |
| Phylum | 38 | 5 |  |
| Class | 81 | 12 |  |
| Order | 180 | 23 |  |
| Family | 364 | 38 |  |
| Genus | 1242 | 110 |  |
| Species | 4483 | 245 |  |
| Number of sequences | 70,606,106 | 8,071,654 |  |
| ⎯⎯⎯⎯⎯⎯⎯⎯⎯⎯⎯⎯⎯⎯⎯⎯⎯⎯⎯⎯⎯⎯⎯⎯⎯⎯⎯⎯⎯⎯⎯⎯⎯⎯⎯⎯ | | | |
