# Supplementary material for: A nitrification bioreactor applied solely with ammonium and inorganic C maintains a highly diverse bacterial and archaeal community even after nine years
Source: Biodegradation. 2026 Jul 6;37(4):111. doi: 10.1007/s10532-026-10288-9 (PMC13337855; doi:10.1007/s10532-026-10288-9)
Supplement: Supplementary file 6 — Supplementary file6 (DOCX 32 KB) [file 10532_2026_10288_MOESM6_ESM.docx]

Table S3. A summary of some the genes detected in the bioreactor involved in nitrogen cycling processes.

| ⎯⎯⎯⎯⎯⎯⎯⎯⎯⎯⎯⎯⎯⎯⎯⎯⎯⎯⎯⎯⎯⎯⎯⎯⎯⎯⎯⎯⎯⎯⎯⎯⎯⎯⎯⎯⎯⎯⎯⎯⎯⎯⎯⎯ | | | |
| --- | --- | --- | --- |
| **Gene** | **Description** | | |
| ⎯⎯⎯⎯⎯⎯⎯⎯⎯⎯⎯⎯⎯⎯⎯⎯⎯⎯⎯⎯⎯⎯⎯⎯⎯⎯⎯⎯⎯⎯⎯⎯⎯⎯⎯⎯⎯⎯⎯⎯⎯⎯⎯⎯ | | | |
| **Nitrification Ammonium monooxygenase: Archaea** | |  |  |
| *amoA* | Ammonia monooxygenase subunit A, partial [Candidatus Nitrosocosmicus arcticus] (<https://www.ncbi.nlm.nih.gov/protein/QED55335.1>) | | |
| *amoB* | Putative archaeal ammonia monooxygenase subunit B [[*Candidatus* Nitrosocaldus yellowstonensis](https://www.uniprot.org/taxonomy/498375)**]** (<https://www.uniprot.org/uniprot/B0LKZ4>) (You et al., 2009, <https://doi.org/10.1016/j.watres.2009.01.016>) | | |
| *amoC* | Ammonia monooxygenase/methane monooxygenase subunit C [uncultured marine Thaumarchaeote SAT1000_12_G09] (<https://www.ncbi.nlm.nih.gov/protein/AIF23051.1>) | | |
| ⎯⎯⎯⎯⎯⎯⎯⎯⎯⎯⎯⎯⎯⎯⎯⎯⎯⎯⎯⎯⎯⎯⎯⎯⎯⎯⎯⎯⎯⎯⎯⎯⎯⎯⎯⎯⎯⎯⎯⎯⎯⎯⎯⎯ | | | |
| **Nitrification Ammonium monooxygenase: Bacteria** | |  |  |
| ⎯⎯⎯⎯⎯⎯⎯⎯⎯⎯⎯⎯⎯⎯⎯⎯⎯⎯⎯⎯⎯⎯⎯⎯⎯⎯⎯⎯⎯⎯⎯⎯⎯⎯⎯⎯⎯⎯⎯⎯⎯⎯⎯⎯ | | | |
| *amoA* | Ammonia monooxygenase alpha subunit. [Nitrosomonas europaea] (strain ATCC 19718)] (<https://www.uniprot.org/uniprot/Q04507>) | | |
| *amoB* | Ammonia monooxygenase beta subunit. [Nitrosomonas europaea] (strain ATCC 19718)] (<https://www.uniprot.org/uniprot/Q04508>) | | |
| *petC* | Ammonia monooxygenase gamma subunit [Nitrosomonas europaea] (strain ATCC 19718)] (<https://www.uniprot.org/uniprotkb/Q82W83>) | | |
| ⎯⎯⎯⎯⎯⎯⎯⎯⎯⎯⎯⎯⎯⎯⎯⎯⎯⎯⎯⎯⎯⎯⎯⎯⎯⎯⎯⎯⎯⎯⎯⎯⎯⎯⎯⎯⎯⎯⎯⎯⎯⎯⎯⎯ | | | |
| **Nitrification** | |  |  |
| ⎯⎯⎯⎯⎯⎯⎯⎯⎯⎯⎯⎯⎯⎯⎯⎯⎯⎯⎯⎯⎯⎯⎯⎯⎯⎯⎯⎯⎯⎯⎯⎯⎯⎯⎯⎯⎯⎯⎯⎯⎯⎯⎯⎯ | | | |
| *hao* | Hydroxylamine oxidoreductase [*Nitrosomonas* mobilis] (<https://www.uniprot.org/uniprot/A0A1G5SG74>) | | |
| *nxrA* | Putative nitrite oxidoreductase, alpha subunit [*Nitrospira japonica*] (<https://www.uniprot.org/uniprot/A0A1W1I298>). (Lücker et al., 2010, <https://doi.org/10.1073/pnas.1003860107>) | | |
| *nxrB* | Putative nitrite oxidoreductase, beta subunit [*Nitrospira japonica*] (<https://www.uniprot.org/uniprot/A0A1W1I5Y1>). (Lücker et al., 2010, <https://doi.org/10.1073/pnas.1003860107>) | | |
| ⎯⎯⎯⎯⎯⎯⎯⎯⎯⎯⎯⎯⎯⎯⎯⎯⎯⎯⎯⎯⎯⎯⎯⎯⎯⎯⎯⎯⎯⎯⎯⎯⎯⎯⎯⎯⎯⎯⎯⎯⎯⎯⎯⎯ | | | |
| **Dissimilatory NO_3_^-^ reductase: membrane bound** | |  |  |
| ⎯⎯⎯⎯⎯⎯⎯⎯⎯⎯⎯⎯⎯⎯⎯⎯⎯⎯⎯⎯⎯⎯⎯⎯⎯⎯⎯⎯⎯⎯⎯⎯⎯⎯⎯⎯⎯⎯⎯⎯⎯⎯⎯⎯ | | | |
| *narB* | Putative respiratory nitrate reductase subunit Rieske. [Haloferax mediterranei] (<https://www.uniprot.org/uniprot/I3R9N2>). (Hidalgo-García et al., 2019, <https://doi.org/10.3389/fmicb.2019.00980>) | | |
| *narC* | Nitrate reductase catalytic subunit [*Streptomyces griseorubens*] (Feng et al., 2014, <https://doi.org/10.1186/s12934-014-0174-4>). Encodes for cytochrome *b-*561 in the dissimilatory nitrate reductase operon of [*Haloarcula marismortui*] (Yoshimatsu et al., 2007, <https://doi.org/10.1007/s00792-006-0016-3>) | | |
| *narG* | Nitrate reductase A subunit alpha [Escherichia coli str. K-12 substr. MG1655]. (<https://www.ncbi.nlm.nih.gov/gene/945782>) | | |
| *narH* | Nitrate reductase subunit beta [Escherichia coli str. K-12 substr. MG1655]. (<https://www.ncbi.nlm.nih.gov/gene/945780>) | | |
| *narI* | Nitrate reductase subunit gamma [Escherichia coli str. K-12 substr. MG1655]. (<https://www.ncbi.nlm.nih.gov/gene/945808>) | | |
| *narJ* | Nitrate reductase 1 molybdenum cofactor assembly chaperone [Escherichia coli str. K-12 substr. MG1655]. (<https://www.ncbi.nlm.nih.gov/gene/945807>) | | |
| *narV* | Nitrate reductase Z subunit gamma [Escherichia coli str. K-12 substr. MG1655]. (<https://www.ncbi.nlm.nih.gov/gene/946029>) | | |
| *narY* | Nitrate reductase Z subunit beta [Escherichia coli str. K-12 substr. MG1655]. (<https://www.ncbi.nlm.nih.gov/gene/946034>) | | |
| *narZ* | Nitrate reductase Z subunit alpha [Escherichia coli str. K-12 substr. MG1655]. (<https://www.ncbi.nlm.nih.gov/gene/945999>) | | |
| ⎯⎯⎯⎯⎯⎯⎯⎯⎯⎯⎯⎯⎯⎯⎯⎯⎯⎯⎯⎯⎯⎯⎯⎯⎯⎯⎯⎯⎯⎯⎯⎯⎯⎯⎯⎯⎯⎯⎯⎯⎯⎯⎯⎯ | | | |
| **Dissimilatory NO_3_^-^ reductase: periplasmic** | |  |  |
| ⎯⎯⎯⎯⎯⎯⎯⎯⎯⎯⎯⎯⎯⎯⎯⎯⎯⎯⎯⎯⎯⎯⎯⎯⎯⎯⎯⎯⎯⎯⎯⎯⎯⎯⎯⎯⎯⎯⎯⎯⎯⎯⎯⎯ | | | |
| *napA* | Periplasmic nitrate reductase subunit NapA [Escherichia coli str. K-12 substr. MG1655]. (<https://www.ncbi.nlm.nih.gov/gene/947093>) | | |
| *napB* | Periplasmic nitrate reductase cytochrome c550 protein [Escherichia coli str. K-12 substr. MG1655]. (<https://www.ncbi.nlm.nih.gov/gene/946698>) | | |
| *napC* | Periplasmic nitrate reductase cytochrome c protein [Escherichia coli str. K-12 substr. MG1655]. (<https://www.ncbi.nlm.nih.gov/gene/946706>) | | |
| ⎯⎯⎯⎯⎯⎯⎯⎯⎯⎯⎯⎯⎯⎯⎯⎯⎯⎯⎯⎯⎯⎯⎯⎯⎯⎯⎯⎯⎯⎯⎯⎯⎯⎯⎯⎯⎯⎯⎯⎯⎯⎯⎯⎯ | | | |
| **Assimilatory NO_3_^-^ reductase** | |  |  |
| ⎯⎯⎯⎯⎯⎯⎯⎯⎯⎯⎯⎯⎯⎯⎯⎯⎯⎯⎯⎯⎯⎯⎯⎯⎯⎯⎯⎯⎯⎯⎯⎯⎯⎯⎯⎯⎯⎯⎯⎯⎯⎯⎯⎯ | | | |
| *nasA* | Nitrate transporter [[*Pseudomonas aeruginosa* PAO1](https://www.ncbi.nlm.nih.gov/Taxonomy/Browser/wwwtax.cgi?id=208964)] (<https://www.ncbi.nlm.nih.gov/gene/877748>)  Assimilatory nitrate reductase [Haloferax mediterranei (strain ATCC 33500)]. (<https://www.uniprot.org/uniprot/I3R634>) | | |
| *nasB* | Assimilatory nitrate reductase (electron transfer subunit NasB) [*Bacillus* *subtilus* subsp. Subtilis str. 168]. (<https://www.ncbi.nlm.nih.gov/gene/938328>)  Nitrite reductase [NAD(P)H] large subunit [Klebsiella oxytoca] (<https://www.uniprot.org/uniprot/Q06458>) | | |
| *narB* | Nitrate reductase. [Synechocystis sp. (strain ATCC 27184)] (<https://www.uniprot.org/uniprot/P73448>). Nitrate reductase electron transfer subunit [*Streptomyces griseorubens*] (Feng et al., 2014, <https://doi.org/10.1186/s12934-014-0174-4>) | | |
| *NR* | In eukaryotes, reduction of nitrate to nitrite is catalyzed by the molybdenum-containing NAD(P)H:nitrate reductase. (Fischer et al., 2005, <https://doi.org/10.1105/tpc.104.029694>) | | |
| ⎯⎯⎯⎯⎯⎯⎯⎯⎯⎯⎯⎯⎯⎯⎯⎯⎯⎯⎯⎯⎯⎯⎯⎯⎯⎯⎯⎯⎯⎯⎯⎯⎯⎯⎯⎯⎯⎯⎯⎯⎯⎯⎯⎯ | | | |
| **Dissimilatory NO_2_^-^ reductase** | | | |
| ⎯⎯⎯⎯⎯⎯⎯⎯⎯⎯⎯⎯⎯⎯⎯⎯⎯⎯⎯⎯⎯⎯⎯⎯⎯⎯⎯⎯⎯⎯⎯⎯⎯⎯⎯⎯⎯⎯⎯⎯⎯⎯⎯⎯ | | | |
| \| *nrfA* \| Cytochrome c552 nitrite reductase [Escherichia coli str. K-12 substr. MG1655]. (<https://www.ncbi.nlm.nih.gov/gene/948571>). \| \| --- \| --- \| \| *nrfC* \| Putative menaquinol-cytochrome c reductase 4Fe-4S subunit [Escherichia coli str. K-12 substr. MG1655]. (<https://www.uniprot.org/uniprot/P0AAK7>) \| | | | |
| ⎯⎯⎯⎯⎯⎯⎯⎯⎯⎯⎯⎯⎯⎯⎯⎯⎯⎯⎯⎯⎯⎯⎯⎯⎯⎯⎯⎯⎯⎯⎯⎯⎯⎯⎯⎯⎯⎯⎯⎯⎯⎯⎯⎯ | | | |
| **Assimilatory NO_2_^-^ reductase** | |  |  |
| ⎯⎯⎯⎯⎯⎯⎯⎯⎯⎯⎯⎯⎯⎯⎯⎯⎯⎯⎯⎯⎯⎯⎯⎯⎯⎯⎯⎯⎯⎯⎯⎯⎯⎯⎯⎯⎯⎯⎯⎯⎯⎯⎯⎯ | | | |
| *nirA* | Nitrite reductase [*Anabaena* sp. Strain PCC 7120] (Frías and Flores, 2015, <https://doi.org/10.1128/jb.00198-15>) | | |
| *nirB* | Assimilatory nitrite reductase large subunit [*Pseudomonas aeruginosa* PAO1] (<https://www.ncbi.nlm.nih.gov/gene/877714>). Nitrite reductase (NADH) large subunit [Escherichia coli str. K-12 substr. MG1655] (<https://www.ncbi.nlm.nih.gov/gene/947868>) | | |
| *nirD* | Nitrite reductase small subunit NirD [*Ectopseudomonas oleovorans* GD03646]. (<https://www.ncbi.nlm.nih.gov/gene/300418522>) | | |
| ⎯⎯⎯⎯⎯⎯⎯⎯⎯⎯⎯⎯⎯⎯⎯⎯⎯⎯⎯⎯⎯⎯⎯⎯⎯⎯⎯⎯⎯⎯⎯⎯⎯⎯⎯⎯⎯⎯⎯⎯⎯⎯⎯⎯ | | | |

Table S2. Continued.

| ⎯⎯⎯⎯⎯⎯⎯⎯⎯⎯⎯⎯⎯⎯⎯⎯⎯⎯⎯⎯⎯⎯⎯⎯⎯⎯⎯⎯⎯⎯⎯⎯⎯⎯⎯⎯⎯⎯⎯⎯⎯⎯⎯⎯ | | |
| --- | --- | --- |
| **Denitrification** | | |
| ⎯⎯⎯⎯⎯⎯⎯⎯⎯⎯⎯⎯⎯⎯⎯⎯⎯⎯⎯⎯⎯⎯⎯⎯⎯⎯⎯⎯⎯⎯⎯⎯⎯⎯⎯⎯⎯⎯⎯⎯⎯⎯⎯⎯ | | |
| *nirK* | Copper-containing nitrite reductase [[*Ralstonia pickettii*]](https://www.ncbi.nlm.nih.gov/Taxonomy/Browser/wwwtax.cgi?id=329) (<https://www.ncbi.nlm.nih.gov/gene/61389570>) | |
| *nirS* | Nitrite reductase [[*Pseudomonas aeruginosa* PAO1](https://www.ncbi.nlm.nih.gov/Taxonomy/Browser/wwwtax.cgi?id=208964)] (<https://www.ncbi.nlm.nih.gov/gene/882217>) | |
| *norB* | Nitric oxide reductase subunit B [[*Pseudomonas aeruginosa* PAO1](https://www.ncbi.nlm.nih.gov/Taxonomy/Browser/wwwtax.cgi?id=208964)] (<https://www.ncbi.nlm.nih.gov/gene/882193>) | |
| *norC* | Nitric oxide reductase subunit C [[*Pseudomonas aeruginosa* PAO1](https://www.ncbi.nlm.nih.gov/Taxonomy/Browser/wwwtax.cgi?id=208964)] (<https://www.ncbi.nlm.nih.gov/gene/882200>) | |
| *nosZ* | Nitrous-oxide reductase (gene ID 879824) [[*Pseudomonas aeruginosa* PAO1](https://www.ncbi.nlm.nih.gov/Taxonomy/Browser/wwwtax.cgi?id=208964)] (<https://www.ncbi.nlm.nih.gov/gene/?term=Pseudomonas+aeruginosa+nosZ>) | |
| ⎯⎯⎯⎯⎯⎯⎯⎯⎯⎯⎯⎯⎯⎯⎯⎯⎯⎯⎯⎯⎯⎯⎯⎯⎯⎯⎯⎯⎯⎯⎯⎯⎯⎯⎯⎯⎯⎯⎯⎯⎯⎯⎯⎯ | | |
| **N_2_ fixation** | |  |
| ⎯⎯⎯⎯⎯⎯⎯⎯⎯⎯⎯⎯⎯⎯⎯⎯⎯⎯⎯⎯⎯⎯⎯⎯⎯⎯⎯⎯⎯⎯⎯⎯⎯⎯⎯⎯⎯⎯⎯⎯⎯⎯⎯⎯ | | |
| *nifD* | Nitrogenase molybdenum-iron protein alpha chain [[*Rhodobacter capsulatus* SB 1003]](https://www.ncbi.nlm.nih.gov/Taxonomy/Browser/wwwtax.cgi?id=272942) (<https://www.ncbi.nlm.nih.gov/gene/31489521>) | |
| *nifH* | Nitrogenase iron protein [*Sinorhizobium meliloti*] (<https://www.ncbi.nlm.nih.gov/gene/89573868>) | |
| ⎯⎯⎯⎯⎯⎯⎯⎯⎯⎯⎯⎯⎯⎯⎯⎯⎯⎯⎯⎯⎯⎯⎯⎯⎯⎯⎯⎯⎯⎯⎯⎯⎯⎯⎯⎯⎯⎯⎯⎯⎯⎯⎯⎯ | | |
| **Anammox** | | |
| ⎯⎯⎯⎯⎯⎯⎯⎯⎯⎯⎯⎯⎯⎯⎯⎯⎯⎯⎯⎯⎯⎯⎯⎯⎯⎯⎯⎯⎯⎯⎯⎯⎯⎯⎯⎯⎯⎯⎯⎯⎯⎯⎯⎯ | | |
| *hzo* | Putative hydrazine oxidoreductase [Uncultured bacterium] (<https://www.uniprot.org/uniparc/UPI0004E02FEB/entry/A0A076E677>) | |
| ⎯⎯⎯⎯⎯⎯⎯⎯⎯⎯⎯⎯⎯⎯⎯⎯⎯⎯⎯⎯⎯⎯⎯⎯⎯⎯⎯⎯⎯⎯⎯⎯⎯⎯⎯⎯⎯⎯⎯⎯⎯⎯⎯⎯ | | |

<https://www.ncbi.nlm.nih.gov/gene/> and <https://www.uniprot.org/uniprot/> visited last on 05 of February 2026.

| ⎯⎯⎯⎯⎯⎯⎯⎯⎯⎯⎯⎯⎯⎯⎯⎯⎯⎯⎯⎯⎯⎯⎯⎯⎯⎯⎯⎯⎯⎯⎯⎯⎯⎯⎯⎯⎯⎯⎯⎯⎯⎯⎯⎯ |
| --- |
