# Supplementary material for: A nitrification bioreactor applied solely with ammonium and inorganic C maintains a highly diverse bacterial and archaeal community even after nine years
Source: Biodegradation. 2026 Jul 6;37(4):111. doi: 10.1007/s10532-026-10288-9 (PMC13337855; doi:10.1007/s10532-026-10288-9)
Supplement: Supplementary file 7 — Supplementary file7 (DOCX 19 KB) [file 10532_2026_10288_MOESM7_ESM.docx]

Table S4. The temporal and spatial coefficient of variation (CV (%): ratio of standard deviation versus mean of the microbial community in a nitrifying laboratory-scale continuous stirred tank after 9 years of similar conditions.

| ⎯⎯⎯⎯⎯⎯⎯⎯⎯⎯⎯⎯⎯⎯⎯⎯⎯⎯⎯⎯⎯⎯⎯⎯⎯⎯⎯⎯⎯⎯⎯⎯⎯⎯⎯ | | | | | | | | |
| --- | --- | --- | --- | --- | --- | --- | --- | --- |
|  | Bacteria | | | Archaea | | |  |  |
| Taxonomic level | | Temporal | Spatial | | Temporal | Spatial | |  |
| ⎯⎯⎯⎯⎯⎯⎯⎯⎯⎯⎯⎯⎯⎯⎯⎯⎯⎯⎯⎯⎯⎯⎯⎯⎯⎯⎯⎯⎯⎯⎯⎯⎯⎯⎯ | | | | | | | | |
| Phylum | | 34 | 10 | | 35 | 14 | |  |
| Class | | 29 | 10 | | 25 | 11 | |  |
| Order | | 25 | 8 | | 24 | 12 | |  |
| Family | | 26 | 9 | | 23 | 18 | |  |
| Genus | | 28 | 14 | | 26 | 17 | |  |
| Species | | 16 ^a^ | 6 ^a^ | | 32 | 22 | |  |
| Mean | | 26 | 10 | | 28 | 16 | |  |
| ⎯⎯⎯⎯⎯⎯⎯⎯⎯⎯⎯⎯⎯⎯⎯⎯⎯⎯⎯⎯⎯⎯⎯⎯⎯⎯⎯⎯⎯⎯⎯⎯⎯⎯⎯ | | | | | | | | |
| ^a^ The 1000 most abundant bacterial groups were considered. | | | | | | | | |
| ⎯⎯⎯⎯⎯⎯⎯⎯⎯⎯⎯⎯⎯⎯⎯⎯⎯⎯⎯⎯⎯⎯⎯⎯⎯⎯⎯⎯⎯⎯⎯⎯⎯⎯⎯ | | | | | | | | |
